# MARCo: An Interactive Web‐Based Tool for Comprehensive Evaluation of HLA Antibody Reactivity in Luminex Single Antigen Bead Assays

**DOI:** 10.1111/tan.71061

**Published:** 2026-11-05

**Authors:** João Henrique Coelho Campos, Gustavo Vinícius Alves, Alberto Cardoso Martins Lima, Maria Gerbase‐DeLima, Renato de Marco

**Affiliations:** ^1^ Instituto de Imunogenética, Associação Fundo de Incentivo à Pesquisa (IGEN/AFIP) São Paulo SP Brazil; ^2^ Departamento de Nefrologia Universidade Federal de São Paulo São Paulo Brazil

**Keywords:** associated antigens, bioinformatics, correlation, HLA antibodies, HLA‐EMMA, luminex single antigen bead assay, SAB

## Abstract

Evaluating HLA antibody reactivity is essential in clinical transplantation, but it can be complicated by analytical issues related to Luminex single‐antigen bead (SAB) assays. These challenges include interpreting mean fluorescence intensity (MFI), variations across platforms and false‐negative and false‐positive reactions. In this regard, we developed the MARCo (Molecular Antigen Reactivity Correlation) platform, an interactive bioinformatic tool designed for multi‐dimensional analysis of HLA antibody reactivity. MARCo is based on a dataset of 70,955 SAB results derived from distinct patients' sera, covering panels from both One Lambda/Thermo Fisher and Immucor/Werfen. In its main screen, MARCo features: (I) A scatter plot displaying MFI data with user‐defined reactivity cutoffs; (II) Details on serological correlation statistics, including the linear equation, *R* ^2^ and Spearman's correlation results; (III) serological assignments using the HATS (HLA Allele to Serotype) methodology; and (IV) quantification of total and solvent‐exposed amino acid mismatches using the HLA‐EMMA algorithm. The tool also provides a Correlation Matrix module that contains interactive heatmaps to visualise reactivity relationships across all HLA loci. By combining correlations with structural and serological data, MARCo enables nuanced interpretation of complex HLA antibody profiles, facilitates investigation of structure‐antigenic relationships and helps clarify platform‐specific assay behaviours. Moreover, MARCo may also be used to explore potential novel determining epitopes to refine the newly approved serological nomenclature further. Altogether, this web platform offers a robust and integrated research environment that combines MFI, serological and molecular data. MARCo platform is freely available for research use only at https://www.igen.org.br/marco/.

**Abbreviations**
DEP: determining epitopes
DSA: donor‐specific antibody
EMMA: epitope mismatch algorithm
HATS: HLA allele to serotype
MARCo: molecular antigen reactivity correlation
MFI: mean fluorescence intensity
SAB: luminex single bead assay

## 1 Introduction

The accurate characterisation of donor‐specific antibodies (DSA) directed against HLA is essential for successful virtual crossmatching, post‐transplant immune monitoring, and guiding clinical decision‐making in solid organ and haematopoietic cell transplantation [1, 2, 3, 4, 5, 6, 7, 8].

Currently, the Luminex single‐antigen bead (SAB) assay is the gold standard for identifying anti‐HLA antibodies due to its high sensitivity and specificity. However, the SAB assay presents important limitations and pitfalls, including false‐negative and false‐positive reactions [9, 10, 11, 12, 13, 14], its semi‐quantitative nature [15] and the difference in mean fluorescence intensity (MFI) across kits from different vendors [16, 17]. Additionally, the limited number of beads in SAB panels restricts a comprehensive representation of HLA allelic diversity.

To facilitate and refine the analysis of SAB results, we developed the MARCo (Molecular Antigen Reactivity Correlation) platform. This interactive bioinformatics tool enables a multi‐dimensional evaluation of HLA antibody reactivity and is built upon a large dataset comprising 70,955 independent SAB results. MARCo is designed to be user‐friendly and is freely available for research use only at https://www.igen.org.br/marco/.

The purpose of this communication is to describe all the features of MARCo and to illustrate some practical applications of the analyses that the software provides.

## 2 Materials and Methods

### 2.1 HLA Antibody Reactivity Dataset

The development of the MARCo platform relied on a retrospective dataset comprising 70,955 independent SAB assays performed in our laboratory, between 2006 and 2025. To avoid bias, the data are limited to one result per patient/class/manufacturer. For example, for an individual with multiple SAB tests for the same class and manufacturer, the most positive serum (e.g., the one with the highest overall sum of MFIs) was chosen for inclusion in the tool. No exclusion criteria were applied to this dataset. All sera were heat‐treated at 56°C for 1 min to eliminate complement interfering factors [18]. Sera with non‐specific background were treated with Fetal Bovine Serum (FBS) [19]. The dataset is stratified by assay manufacturer and HLA class: 52,812 One Lambda/Thermo Fisher (Canoga Park, CA) Standard assays, 16,056 Immucor/Werfen (Norcross, GA) assays, and 2087 One Lambda/Thermo Fisher ExPlex assays. Each entry included MFI values linked to anonymised clinical metadata, including sex and history of sensitising events (transfusions, transplants and pregnancies), enabling stratified downstream analyses.

### 2.2 Data Processing and Harmonisation Pipeline

Data ingestion, cleaning and transformation were executed using an in‐house custom pipeline developed in Python (v3.12), utilising the Polars (v1.31.0) and PyArrow (v18.0.0) libraries for high‐performance data manipulation. While One Lambda/Thermo Fisher software exports background‐adjusted data, Immucor/Werfen software typically provides raw fluorescence values. To address this, a locus‐specific baseline correction algorithm was applied to all Immucor/Werfen samples. For each HLA locus within a sample, the minimum MFI value was identified as the baseline and subtracted from all other alleles at that locus. This normalisation process enabled the unified analysis of reactivity patterns across different platforms.

### 2.3 Molecular and Serological Characterisation

To provide a comprehensive environment for interpreting HLA antibody reactivity, the MARCo platform integrates external tools. Associated antigens (previously classified as serotypes) were determined using the HATS (HLA Allele to Serotype) methodology [20, 21]. This mapping is based on critical amino acid substitutions at determining epitopes (DEPs) defined in the IPD‐IMGT/HLA Database. Additionally, the HLA‐EMMA algorithm (v1.06) [22] was used to evaluate the structural dissimilarities between allele pairs by calculating both the total number of amino acid mismatches and the specific count of solvent‐exposed residues, which are accessible for antibody binding and are therefore more relevant to immunogenicity.

### 2.4 Statistical Analysis

The statistical relationship between the reactivities of allele pairs in SAB was assessed using the Spearman rank correlation coefficient (ρ) as the main metric. This non‐parametric method was chosen for its effectiveness in managing non‐linear distributions and the frequent outliers typically found in MFI datasets. Pearson correlation coefficients were calculated secondarily to determine the coefficients of determination ($R^{2}$) for variance analysis. All statistical calculations included significance testing and documentation of sample sizes to ensure the reliability of the observed correlations.

## 3 Results

### 3.1 Platform Architecture and Analytical Scope

MARCo was developed as an interactive web‐based dashboard designed to dissect the complexity of HLA antibody reactivity through two primary analytical modules: a pairwise Scatter Plot interface for detailed allele comparison and a Correlation Matrix interface for global pattern recognition. It is important to note that the platform's analytical scope is intrinsically defined by the composition of the commercial SAB kits used to generate the source dataset; consequently, users can only explore combinations of alleles that are physically present in the One Lambda/Thermo Fisher or Immucor/Werfen panels. The following sections describe representative analyses—using HLA‐B (Class I) for pairwise comparison and HLA‐DQB1 (Class II) for matrix analysis—to illustrate the platform's capabilities in integrating statistical, serological and molecular data.

While the figures focus on representative analyses, users are encouraged to explore the MARCo tool further by systematically adjusting the interactive filters and visualisation parameters available in each module. This design supports sensitivity analyses around MFI cutoffs, manufacturer‐ and kit‐specific behaviour and clinically relevant stratifications (e.g., sex and sensitising events), enabling users to generate and test hypotheses directly within the interface.

### 3.2 Pairwise Reactivity and Molecular Integration: A Class I Example

Figure 1 illustrates the MARCo interface, using a representative pairwise analysis of HLA‐B*35:01 and HLA‐B*53:01. The tool interface is organised into four quadrants: the *Scatter Plot* (Figure 1A) displays the MFI values for both alleles. Blue dots indicate concordant samples, while red dots represent discordant samples based on user‐defined thresholds (Positive > 1,500; Negative < 300). The *Results* section (Figure 1B) provides the linear regression equation, the coefficient of determination (*R* ^2^), the Spearman correlation (ρ), and quantifies the counts and percentages of discordant samples (those positive for one allele and negative for the other). *Serological Classification* (Figure 1C) lists the HLA‐associated antigen designation, its qualifier, and the broad and Bw4/6 classifications. The *Amino Acid Mismatches* section (Figure 1D) details the total and solvent‐accessible amino acid differences between the selected pair as determined by HLA‐EMMA.

**FIGURE 1:**
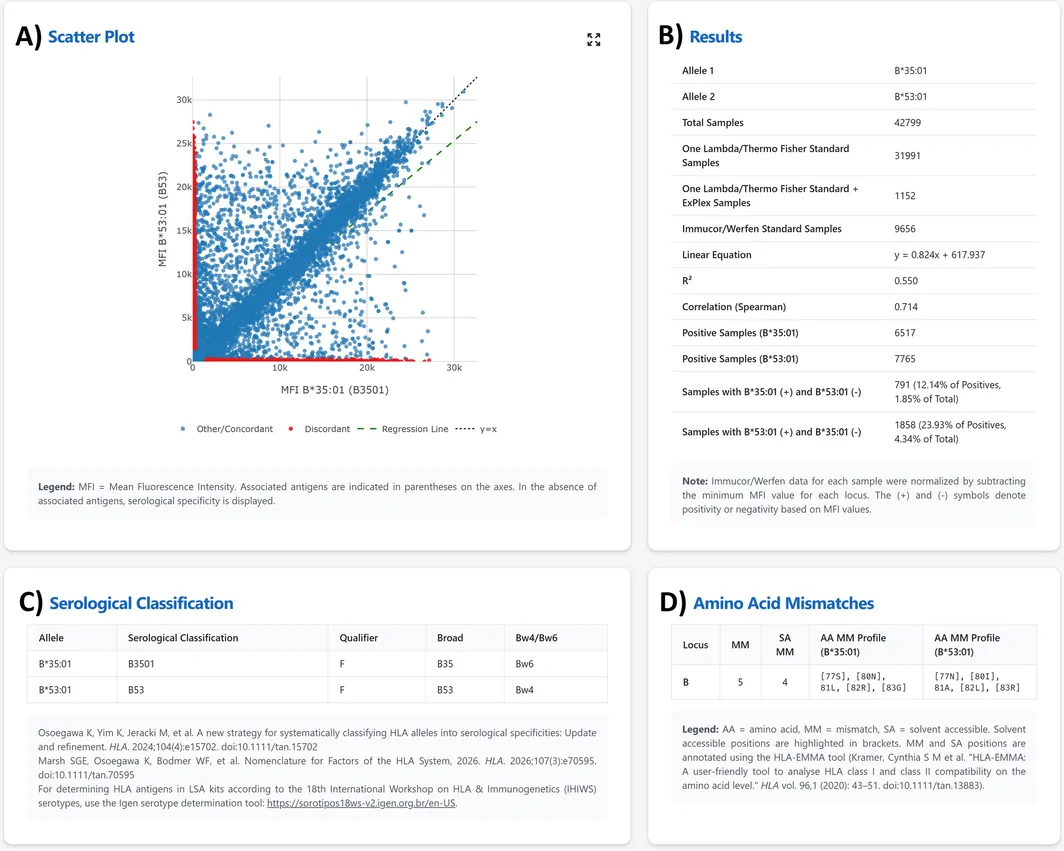
Representative pairwise reactivity analysis of Class I alleles using the Scatter Plot module. The interface is shown analysing the relationship between HLA‐B*35:01 and HLA‐B*53:01 (*n* = 42,799 samples). Scatter Plot (A): The visualisation displays mean fluorescence intensity (MFI) values for both alleles. Blue dots represent concordant samples, while red dots highlight discordant samples based on user‐defined cutoffs (positive > 1,500; negative < 300). Results (B): The side panel displays the linear regression equation, coefficient of determination (*R* ^2^), Spearman correlation (ρ), and quantifying counts and percentages of discordant samples (positive for one allele and negative for the other). The lower panels present the ‘Serological Classification’ (C) and the ‘Amino Acid Mismatches’ (D) table (HLA‐EMMA) listing the total and solvent‐accessible amino acid differences between the selected pair.

The Scatter Plot module facilitates direct comparison of MFI values between any two selected alleles, enabling dissection of cross‐reactivity versus allele‐specific recognition. The representative analysis of HLA‐B*35:01 and HLA‐B*53:01 illustrates the depth of data integration (Figure 1). To accommodate the lack of standardised positivity thresholds across laboratories, the interface includes dynamic sliders that let users define custom MFI cutoffs for positive and negative classifications. In this specific Class I scenario, the tool calculated a Spearman correlation of ρ = 0.714. However, the integrated statistical panel revealed a significant bifurcation in reactivity: 1858 samples (4.34% of the total) were positive for B*53:01 but negative for B*35:01, while 791 samples (1.85%) showed the inverse pattern. To elucidate the biological basis of this discordance, MARCo automatically retrieved the serological and molecular context. The ‘Associated Antigens’ table, derived from HATS, indicates that although both alleles belong to the broad B53/B35 antigen groups, B*53:01 carries the Bw4 epitope, while B*35:01 carries the Bw6 epitope. Concurrently, the ‘Mismatches’ table, powered by HLA‐EMMA, identified five amino acid differences between the pair, four of which are solvent‐accessible. This integration enables the researcher to directly link the observed statistical discordance (red points in the scatter plot) to specific molecular targets (e.g., Bw4/Bw6 epitopes or specific solvent‐exposed residues).

Beyond the representative example shown in Figure 1, the Scatter Plot module includes a comprehensive set of interactive filters and display features to encourage exploratory analysis across the full space of alleles available in the commercial panels. Users can select any two alleles present in the dataset and perform sensitivity analyses by adjusting MFI cutoffs for both positivity and negativity using sliders that span 0–5000 MFI (with default thresholds of > 1500 for positive and < 300 for negative). The dataset can be filtered by manufacturer (One Lambda/Thermo Fisher, Immucor/Werfen, or All Manufacturers). When One Lambda/Thermo Fisher is selected, an additional drop‐down menu becomes available to select one or multiple lots of interest. Kit‐level filtering is also supported, allowing selection of Standard kits for both manufacturers, and (for One Lambda/Thermo Fisher) the additional option of ‘Standard + ExPlex’.

Clinical stratification filters allow users to subset samples by sex and sensitising history. Samples can be stratified by sex, by number of transfusions (all, 0, 1–5 and > 5), and by number of transplants (all, 0 or ≥ 1). When female samples are selected, the pregnancy filter is enabled (all, 0 or ≥ 1), supporting targeted analyses of pregnancy‐associated sensitisation. The scatter plot is responsive and can be explored in detail via zoom, facilitating inspection of dense regions and boundary behaviour near selected cutoffs. Each point is interactive, displaying a tooltip that includes the MFI for each selected allele, an anonymised Sample ID, and the associated manufacturer and kit. In the context of Class II, where heterodimer notation is important, if either DQB1 or DPB1 is selected, the alleles are presented as heterodimers, displaying both beta and alpha chains.

### 3.3 Global Correlation Patterns and Serological Clustering: A Class II Example

The Correlation Matrix module extends the analysis to a macroscopic level, generating interactive heatmaps that visualise reactivity relationships across entire loci. A representative analysis of the HLA‐DQB1 locus (Figure 2) demonstrates the tool's ability to uncover global serological patterns. Users can customise these matrices by selecting specific loci and applying correlation thresholds to filter out weak associations. Zoom functionality allows for the detailed inspection of dense correlation blocks within large matrices.

**FIGURE 2:**
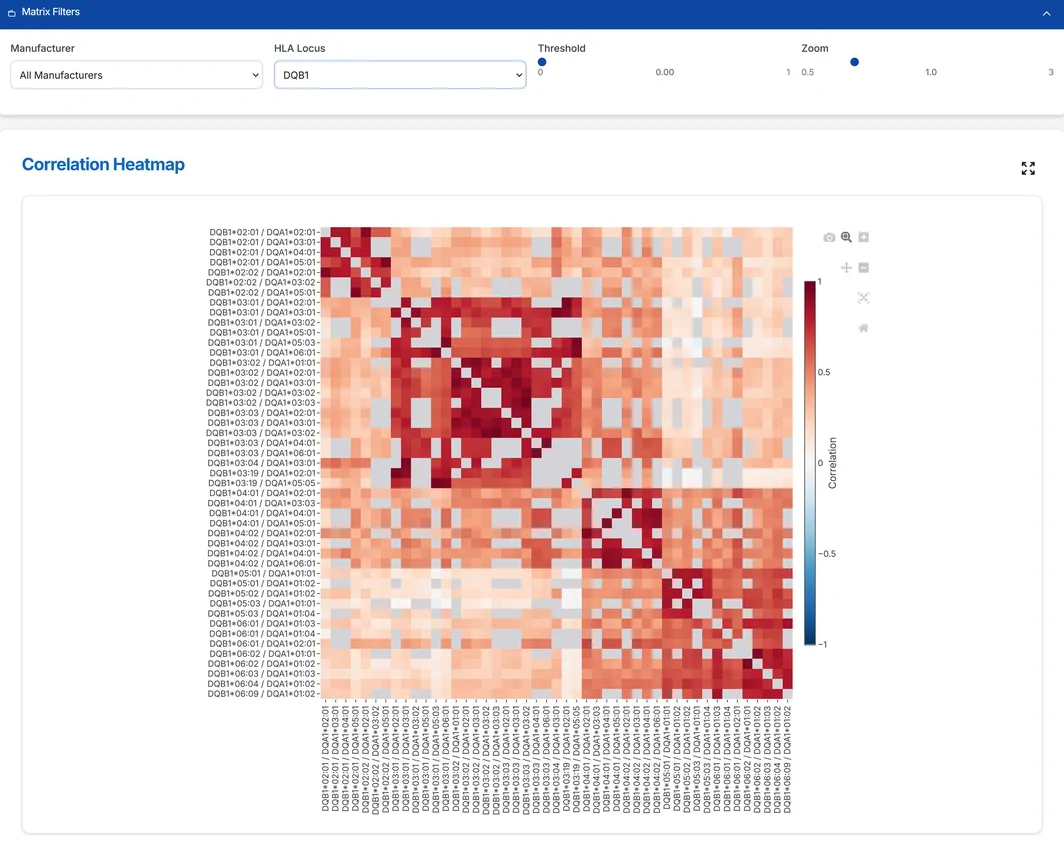
Representative global analysis of Class II alleles using the Correlation Matrix module. The figure displays a heatmap generated for the HLA‐DQB1 locus, utilising the combined dataset from all manufacturers. The axes list DQB1 alleles paired with their associated DQA1 chains. The colour scale represents the strength of the Spearman correlation, ranging from dark blue (−1) to dark red (+1). High‐density clusters of positive correlation are visible along the diagonal, corresponding to serologically defined groups such as DQ2 (top left), the DQ3 broad (centre), and DQ1 broad (bottom right). The interface header shows active filters for ‘All Manufacturers’ and the specific selection of the ‘DQB1’ locus.

The heatmap generated for HLA‐DQB1 effectively reproduces established serological patterns without the need for prior labelling or manual grouping. The visualisation reveals high‐density correlation clusters along the diagonal that align with classical biological Cross‐Reactive Groups (CREGs). For instance, distinct clusters corresponding to the DQ2‐associated antigen (e.g., DQB1*02 associated with DQA1*02/03/05), the DQ3 broad antigen group (encompassing DQ7, DQ8 and DQ9) and the DQ1 broad group (comprising DQ5 and DQ6 splits) are clearly discernible. This visual clustering confirms that the correlations derived from the MFI dataset are consistent with known serological specificities. By toggling the threshold slider, users can further refine these clusters to visualise only the strongest associations, facilitating the identification of potentially redundant beads or tightly linked epitope groups. The other Class I and II HLA heatmaps are included in the Supplementary Figures 1–7.

In addition to the representative DQB1 analysis shown in Figure 2, the Correlation Matrix module provides a structured set of filters that allow users to generate different matrices depending on the subset of data being queried. The Manufacturer filter supports matrix generation for All Manufacturers, One Lambda/Thermo Fisher or Immucor/Werfen, enabling direct comparison of correlation structure across platforms and within manufacturer‐specific subsets. Users can select from multiple matrix types via the HLA Locus filter, including Class I loci (HLA‐A, ‐B or ‐C), Class II loci (HLA‐DRB, ‐DQB1 or ‐DPB1) and inter‐locus matrices for broader pattern discovery (Inter‐locus Class I and Inter‐locus Class II). Within the DRB grouping, matrices can be examined for HLA‐DRB1 and DRB3/DRB4/DRB5, while DQB1 and DPB1 are displayed as heterodimers, reflecting paired beta and alpha chains.

Matrices are fully interactive: users can zoom to inspect dense blocks and local structures, and a Threshold control (Spearman ρ slider) can be applied to display only heatmap cells with correlations meeting or exceeding the selected value, thereby filtering out weaker associations and highlighting the most informative relationships. Figure 3 demonstrates an additional feature of the MARCo Tool: the examination of cross‐reactivity patterns among Classes I and II HLA heatmaps by setting a specific threshold, such as 0.50. For example, at the 0.50 threshold, the HLA‐A heatmap clearly identifies the A2/28 cross‐reactive pattern (Figure 3A). Similarly, using the same threshold, the HLA‐DR heatmap reveals the DR3/5/6/8/52 cross‐reactive pattern (Figure 3B), which is associated with the 96H putative epitope.

**FIGURE 3:**
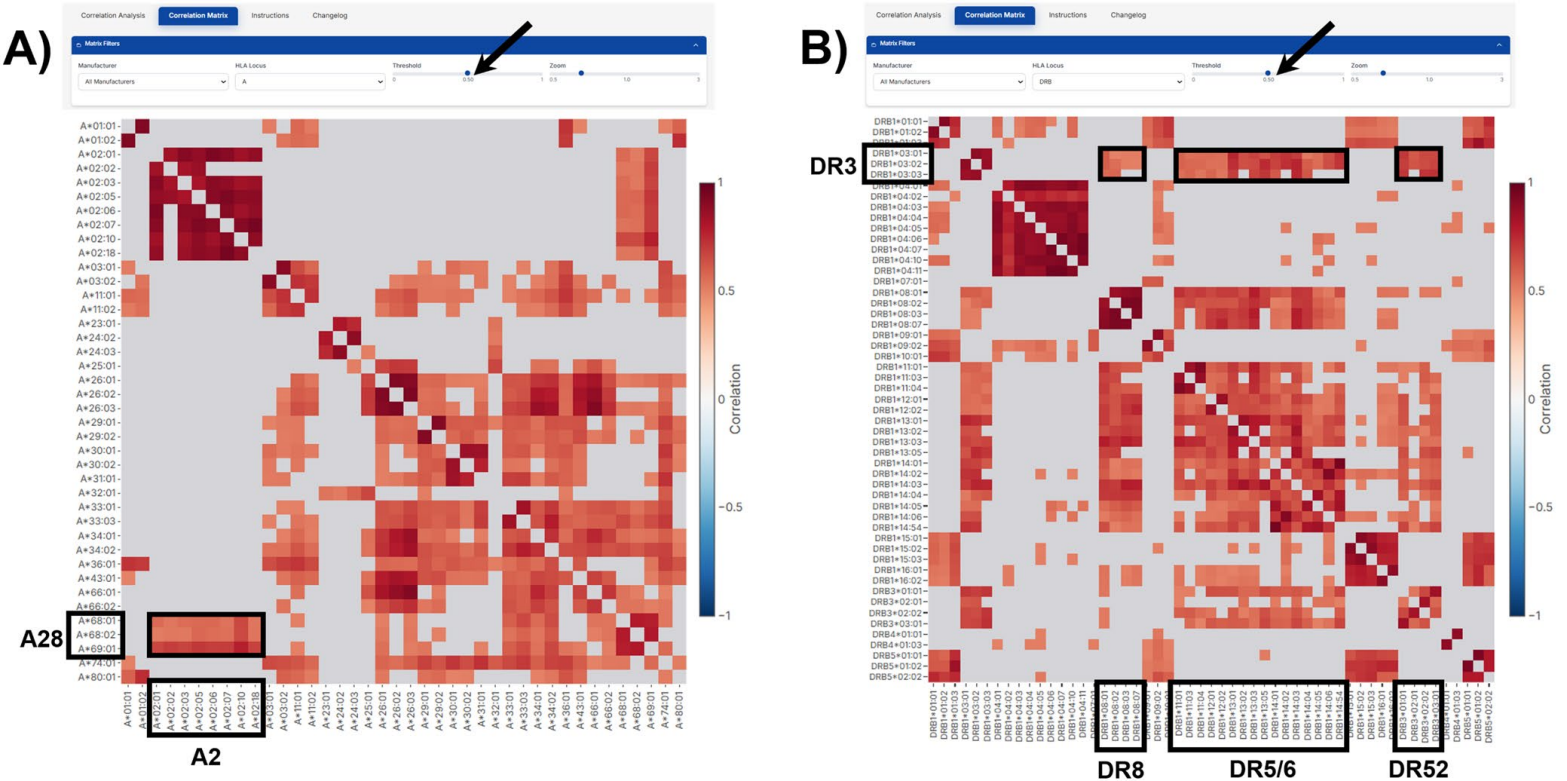
Visualisation of HLA cross‐reactivity patterns using the MARCo interactive correlation matrix. In the correlation matrix, users can set a threshold (e.g., 0.50) to examine cross‐reactivity patterns among Classes I and II HLA heatmaps. (A) At the 0.50 threshold (green arrow), the HLA‐A heatmap shows the A2/28 cross‐reactive pattern (green box). (B) The HLA‐DR heatmap also reveals the DR3/5/6/8/52 cross‐reactive pattern (green boxes) at the same threshold.

Each heatmap cell can be examined interactively, and a tooltip provides the corresponding allele pair, the Spearman correlation coefficient, the number of positive samples for each pair using MARCo's default cutoff (MFI > 1500), the total number of samples contributing to the allele pair combination (stratified by manufacturer and kit), and the associated linear equation.

### 3.4 High‐Correlation Allele Pairs Within Serological Groups

The platform's analytical tools enable the systematic identification of allele pairs exhibiting high correlation (ρ ≈ 0.90 or *R* ^*2*^ ≈ 0.95) within same serological groups. Representative examples across both commercial platforms demonstrate nearly identical antibody reactivity profiles throughout the entire quantitative MFI range (Figure 4). In Class I, high correlation is observed for A*02:01/A*02:06 (One Lambda/Thermo Fisher) and A*02:02/A*02:05 (Immucor/Werfen). Similar patterns persist in Class II, specifically for DRB4*01:01/DRB4*01:03 in One Lambda/Thermo Fisher data and DRB1*03:02/DRB1*03:03 in Immucor/Werfen panels.

**FIGURE 4:**
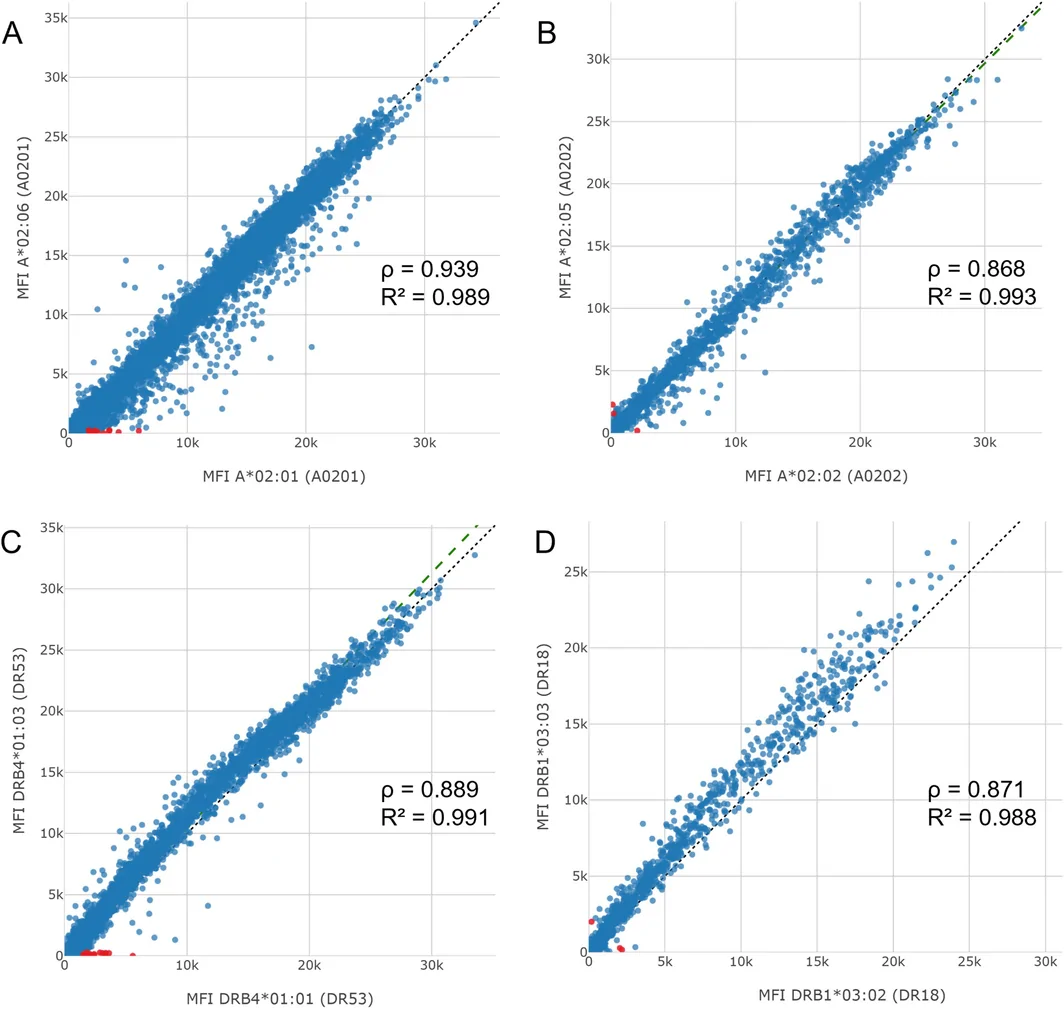
Representative high‐correlation allele pairs within serological groups across commercial SAB platforms. The scatter plots illustrate the highly correlated reactivity patterns of alleles within the same serological groups across different platforms. (A) Comparison between A*02:01 and A*02:06 in One Lambda/Thermo Fisher kit. (B) Comparison between A*02:02 and A*02:05 in Immucor/Werfen kit. (C) Comparison between DRB4*01:01 and DRB4*01:03 in One Lambda/Thermo Fisher kit. (D) Comparison between DRB1*03:02 and DRB1*03:03 in Immucor/Werfen kit. Blue dots represent concordant samples, while red dots indicate discordance based on standard MFI cutoffs. The dashed black line represents the identity line (y = x), and the green dashed line represents the linear regression.

### 3.5 Documentation and Technical Specifications

To ensure the correct interpretation of these high‐dimensional data, the platform includes a dedicated ‘Instructions’ module. This section provides comprehensive documentation on the dataset composition, detailing sample sizes stratified by manufacturer and HLA class. It also elucidates the normalisation algorithms applied to Immucor/Werfen data and the specific methodologies used for serological mapping and molecular mismatch calculation, serving as a technical reference for the platform's analytical backend.

## 4 Discussion

MARCo addresses the interpretative challenges of SAB assays by moving beyond isolated MFI values. While previous literature has extensively documented the limitations of MFI—ranging from lack of standardisation to saturation effects [15]—our integrated analysis of nearly 71,000 samples demonstrates that data‐driven patterns, when contextualised with molecular and serological data, can help in distinguishing biologically relevant reactivity from technical noise.

The MARCo tool can also serve as a reliable quality‐control tool for identifying platform‐specific artefacts in SAB. In routine laboratory practice, one often encounters ‘unexpected’ positive beads that do not fit classical serological patterns. A practical example in this regard involves the non‐specific background reactivity frequently observed against DRB1*01 (DR1), DRB1*10 (DR10) and DRB1*12 (DR12) beads in specific One Lambda/Thermo Fisher SAB lots (e.g., Lot 17). By using MARCo to analyse these alleles, a given user can observe that true anti‐DR1 antibodies typically show a tight correlation between DRB1*01:01 and DRB1*01:02. If a patient sample exhibits isolated positivity for DRB1*01:01 without the corresponding reaction to DRB1*01:02 or vice versa (e.g., discordant reactivity pattern), the user may interpret this as a potential SAB artefact rather than true alloimmunisation. In our laboratory, such observations support the implementation of assay modifications, such as using FBS in the patient's sera, which effectively eliminates this specific background noise [19].

It is worth noting that the MARCo platform facilitates systematic analyses of correlation profiles, identifying HLA allele pairs that exhibit high correlation (e.g., ρ ≈ 0.90 or *R* ^*2*^ ≈ 0.95) within the same serological groups, as illustrated in Figure 3. Indeed, these highly correlated pairs, such as A*02:01/A*02:06, A*02:02/A*02:05, DRB4*01:01/DRB4*01:03 and DRB1*03:02/DRB1*03:03, suggest that many beads in current commercial SAB panels are redundant. As these pairs show almost identical antibody binding profiles, they provide minimal information for differentiating complex antibody patterns. In addition, the presence of multiple beads with very similar reactivity profiles represents a missed opportunity to allocate bead positions to other alleles currently absent from standard panels, such as A*02:11, B*39:10 and DRB1*11:02, thereby expanding epitope coverage and improving the detection of alloantibodies in diverse ethnic individuals. By utilising data‐driven insights from MARCo, manufacturers can optimise panel composition by replacing highly correlated beads with underrepresented alleles, thereby improving the diagnostic utility and global applicability of SAB assays.

Although MARCo provides robust, data‐driven insights, users should be aware that the platform relies on the allele combinations in existing commercial SAB kits. Accordingly, this reliance restricts the exploration of rare HLA variants. Future updates are expected to include machine learning models that can predict the immunogenicity of specific amino acid mismatches. This upcoming advancement will help connect MFI data with clinical decision‐making in transplantation settings.

Some limitations on MARCo should be acknowledged. We cannot rule out that the SAB reactivity dataset includes antibodies against cryptic epitopes on denatured HLA antigens on the beads. This limitation will be addressed in upcoming versions of MARCo by including a dataset of acid‐treated SAB to account for such assay artefacts. In addition, data on sensitising events were gathered by external sources and later reported to our laboratory. Accordingly, we cannot assume they are completely accurate. Lastly, the most recent SAB lots still represent only a small fraction of the MARCo dataset, which may limit its applicability for HLA antibody evaluation in current commercial SAB lots.

In conclusion, MARCo offers a user‐friendly web‐based platform that integrates HLA antibody reactivity, serological correlation, and molecular mismatch assessment. With its comprehensive features, MARCo provides a new perspective on analysing HLA antibodies by converting a static list of MFI values into an interconnected, dynamic framework. As this tool emphasises the unique behaviours of Class I and Class II HLA antibodies, it helps laboratories filter out technical artefacts and enhance their Virtual Crossmatch strategies. Ultimately, MARCo encourages a shift from a solely MFI‐centric interpretation of SAB data to a more nuanced understanding of the structural and serological aspects of humoral responses. The platform is freely accessible in both English and Portuguese for research use only at https://www.igen.org.br/marco/.

## Author Contributions

João H. C. Campos and Gustavo V. Alves developed the tool; João H. C. Campos wrote the initial draft of the manuscript; Alberto C. M. Lima and Maria Gerbase‐De Lima reviewed the manuscript; Renato de Marco designed the study and reviewed the manuscript. All authors have read and approved the final version of the manuscript.

## Funding

This work was supported by Associação Fundo de Incentivo à Pesquisa. CAPES, Finance Code 001.

## Ethics Statement

The study protocol was approved by the Institutional Ethics Committee of the Hospital Maternidade Leonor Mendes de Barros (Approval Number: 8.386.993; CAAE: 97233626.1.0000.0063). The requirement for informed consent was waived due to the retrospective nature of the study and the use of fully de‐identified secondary data.

## Conflicts of Interest

The authors declare no conflicts of interest.

## Supporting information


**Supplementary Figure 1:** HLA‐A correlation heatmap.
**Supplementary Figure 2:** HLA‐B correlation heatmap.
**Supplementary Figure 3:** HLA‐C correlation heatmap.
**Supplementary Figure 4:** HLA‐DR correlation heatmap.
**Supplementary Figure 5:** HLA‐DP correlation heatmap.
**Supplementary Figure 6:** Class I HLA correlation heatmap.
**Supplementary Figure 7:** Class II HLA correlation heatmap.

## Data Availability

The data that support the findings of this study are available from the corresponding author upon reasonable request.

## References

[1] J. Kobashigawa , M. G. Crespo‐Leiro , S. M. Ensminger , et al., “Report From a Consensus Conference on Antibody‐Mediated Rejection in Heart Transplantation,” Journal of Heart and Lung Transplantation 30, no. 3 (2011): 252–269, 10.1016/j.healun.2010.11.003. PMC3829685 21300295

[2] A. Zachary and N. L. Reinsmoen , “Quantifying HLA‐Specific Antibodies in Patients Undergoing Desensitization,” Current Opinion in Organ Transplantation 16, no. 4 (2011): 410–415, 10.1097/MOT.0b013e32834899b8. 21666475

[3] A. Loupy , G. S. Hill , and S. C. Jordan , “The Impact of Donor‐Specific Anti‐HLA Antibodies on Late Kidney Allograft Failure,” Nature Reviews. Nephrology 8, no. 6 (2012): 348–357, 10.1038/nrneph.2012.81. 22508180

[4] B. D. Tait , C. Süsal , H. M. Gebel , et al., “Consensus Guidelines on the Testing and Clinical Management Issues Associated With HLA and Non‐HLA Antibodies in Transplantation,” Transplantation 95, no. 1 (2013): 19–47, 10.1097/TP.0b013e31827a19cc. 23238534

[5] N. M. Valenzuela and E. F. Reed , “Antibody‐Mediated Rejection Across Solid Organ Transplants: Manifestations, Mechanisms, and Therapies,” Journal of Clinical Investigation 127, no. 7 (2017): 2492–2504, 10.1172/JCI90597. PMC5490786 28604384

[6] A. C. M. Lima , C. Bonfim , J. Getz , et al., “Untreated Donor‐Specific HLA Antibodies Are Associated With Graft Failure and Poor Survival After Haploidentical Transplantation With Post‐Transplantation Cyclophosphamide in Pediatric Patients With Nonmalignant Disorders,” Transplantation and Cellular Therapy 28, no. 10 (2022): 698.e1–698.e11, 10.1016/j.jtct.2022.07.019. 35882362

[7] A. C. M. Lima , J. Getz , G. B. do Amaral , et al., “Donor‐Specific HLA Antibodies Are Associated With Graft Failure and Delayed Hematologic Recovery After Unrelated Donor Hematopoietic Cell Transplantation,” Transplantation and Cellular Therapy 29, no. 8 (2023): 493.e1–493.e10, 10.1016/j.jtct.2023.05.014. 37220839

[8] H. C. Sullivan , A. Jaramillo , S. Gaitonde , et al., “Virtual Crossmatching: Principles, Practices, and the Path Forward—An American Society for Histocompatibility and Immunogenetics/College of American Pathologists Collaborative,” American Journal of Transplantation 25, no. 10 (2025): 2048–2056, 10.1016/j.ajt.2025.06.018. 40550468

[9] L. E. Morales‐Buenrostro , P. I. Terasaki , L. A. Marino‐Vázquez , J. H. Lee , N. El‐Awar , and J. Alberú , ““Natural” Human Leukocyte Antigen Antibodies Found in Nonalloimmunized Healthy Males,” Transplantation 86, no. 8 (2008): 1111–1115, 10.1097/TP.0b013e318186d87b. 18946350

[10] M. Schnaidt , C. Weinstock , M. Jurisic , B. Schmid‐Horch , A. Ender , and D. Wernet , “HLA Antibody Specification Using Single‐Antigen Beads–A Technical Solution for the Prozone Effect,” Transplantation 92, no. 5 (2011): 510–515, 10.1097/TP.0b013e31822872dd. 21869744

[11] P. C. Grenzi , R. de Marco , R. Z. R. Silva , E. F. Campos , and M. Gerbase‐DeLima , “Antibodies Against Denatured HLA Class II Molecules Detected in Luminex‐Single Antigen Assay,” Human Immunology 74, no. 10 (2013): 1300–1303, 10.1016/j.humimm.2013.06.035. 23831256

[12] C. L. Dean , S. M. Krummey , H. M. Gebel , R. A. Bray , and H. C. Sullivan , “Identification of a Recurrent Pattern of False‐Positivity by Luminex HLA MHC Class I Single Antigen Bead Testing,” Human Immunology 81, no. 2–3 (2020): 73–78, 10.1016/j.humimm.2019.12.006. PMC7357571 31917024

[13] C. Wehmeier , G. Hönger , and S. Schaub , “Caveats of HLA Antibody Detection by Solid‐Phase Assays,” Transplant International: Official Journal of the European Society for Organ Transplantation 33, no. 1 (2020): 18–29, 10.1111/tri.13484. 31359501

[14] I. H. Noronha , M. Gebarse‐DeLima , L. Z. M. F. Bottino , A. C. M. Lima , R. Fantini , and R. de Marco , “False Positive Reaction Against 73ID EPLET in Single Antigen Bead Assay,” Human Immunology 87, no. 5 (2026): 111708, 10.1016/j.humimm.2026.111708. 41797096

[15] H. C. Sullivan , R. S. Liwski , R. A. Bray , and H. M. Gebel , “The Road to HLA Antibody Evaluation: Do Not Rely on MFI,” American Journal of Transplantation: Official Journal of the American Society of Transplantation and the American Society of Transplant Surgeons 17, no. 6 (2017): 1455–1461, 10.1111/ajt.14229. PMC8663029 28199773

[16] E. F. Reed , P. Rao , Z. Zhang , et al., “Comprehensive Assessment and Standardization of Solid Phase Multiplex‐Bead Arrays for the Detection of Antibodies to HLA,” American Journal of Transplantation 13, no. 7 (2013): 1859–1870, 10.1111/ajt.12287. PMC3967448 23763485

[17] G. E. Karahan , G. W. Haasnoot , K. Voogt‐Bakker , F. H. J. Claas , D. Roelen , and S. Heidt , “A Modeling Approach for Mean Fluorescence Intensity Value Harmonization and Cutoff Prediction for Luminex Single Antigen Bead Assays of Two Different Vendors,” HLA 102, no. 5 (2023): 557–569, 10.1111/tan.15082. 37130801

[18] R. de Marco , A. Goncalves‐Primo , É. F. Campos , and M. Gerbase‐DeLima , “29‐P Heating the Serum at 56°C for One Minute Solves the Problem of Inhibitory Factors in the Luminex Antibody Detection Assays,” Human Immunology 72 (2011): S33, 10.1016/j.humimm.2011.07.052.

[19] K. Jung , M. Nam , Y. J. Hong , et al., “Removal of Non‐Specific Reactions of LABScreen Single Antigen Class II Assay by Fetal Bovine Serum Treatment,” Annals of Clinical and Laboratory Science 49, no. 5 (2019): 678–681. 31611215

[20] K. Osoegawa , S. G. E. Marsh , R. Holdsworth , et al., “A New Strategy for Systematically Classifying HLA Alleles Into Serological Specificities,” HLA 100, no. 3 (2022): 193–231, 10.1111/tan.14662. 35538616

[21] S. G. E. Marsh , K. Osoegawa , W. F. Bodmer , et al., “Nomenclature for Factors of the HLA System, 2026,” HLA 107, no. 3 (2026): e70595, 10.1111/tan.70595. PMC12936402 41742599

[22] C. S. M. Kramer , J. Koster , G. W. Haasnoot , D. L. Roelen , F. H. J. Claas , and S. Heidt , “HLA‐EMMA: A User‐Friendly Tool to Analyse HLA Class I and Class II Compatibility on the Amino Acid Level,” HLA 96, no. 1 (2020): 43–51, 10.1111/tan.13883. PMC7317360 32227681

