# Supplementary material for: MARCo: An Interactive Web‐Based Tool for Comprehensive Evaluation of HLA Antibody Reactivity in Luminex Single Antigen Bead Assays
Source: HLA. 2026 Nov 5;108(5):e71061. doi: 10.1111/tan.71061 (PMC13740467; doi:10.1111/tan.71061)
Supplement: Supplementary file 1 [file TAN-108-e71061-s001.docx]

**MARCo: An interactive web-based tool for comprehensive evaluation of HLA antibody reactivity in Luminex Single Antigen Bead Assays**

**Supplementary Material**

**Supplementary Figure 1. HLA-A correlation heatmap.**

**Supplementary Figure 2. HLA-B correlation heatmap.**

**Supplementary Figure 3. HLA-C correlation heatmap.**

**Supplementary Figure 4. HLA-DR correlation heatmap**

**Supplementary Figure 5. HLA-DP correlation heatmap**

**Supplementary Figure 6. Class I HLA correlation heatmap**

**Supplementary Figure 7. Class II HLA correlation heatmap**
